# Age-Related Effects on the Spectrum of Cerebral Visual Impairment in Children With Cerebral Palsy

**DOI:** 10.3389/fnhum.2022.750464

**Published:** 2022-03-02

**Authors:** Jessica Galli, Erika Loi, Anna Molinaro, Stefano Calza, Alessandra Franzoni, Serena Micheletti, Andrea Rossi, Francesco Semeraro, Elisa Fazzi, CP Collaborative Group

**Affiliations:** ^1^Department of Clinical and Experimental Sciences, University of Brescia, Brescia, Italy; ^2^Unit of Child Neurology and Psychiatry, ASST Spedali Civili of Brescia, Brescia, Italy; ^3^Department of Molecular and Translational Medicine, University of Brescia, Brescia, Italy; ^4^BDbiomed, BODaI Lab, University of Brescia, Brescia, Italy; ^5^Department of Neurological and Vision Sciences, ASST Spedali Civili of Brescia, Brescia, Italy; ^6^Eye Clinic, University of Brescia, Brescia, Italy

**Keywords:** cerebral visual impairment, cognitive-visual disorders, cerebral palsy, age, children

## Abstract

**Background:**

Cerebral Visual Impairment (CVI) is a very common finding in children affected by Cerebral Palsy (CP). In this paper we studied the characteristics of CVI of a large group of children with CP and CVI, describing their neurovisual profiles according to three different age subgroups (subgroup 1: infants 6 months–2 years; subgroup 2: pre-school age 3–5 years; subgroup 3: school age ≥ 6 years).

**Methods:**

We enrolled 180 subjects (104 males, mean age 66 ± 42.6 months; range 6–192 months) with CP and CVI for the study. We carried out a demographic and clinical data collection, neurological examination, developmental or cognitive assessment, and a video-recorded visual function assessment including an evaluation of ophthalmological characteristics, oculomotor functions, and basic visual functions. In school-aged children, we also performed an evaluation of their cognitive-visual profiles.

**Results:**

There were signs of CVI in all the three subgroups. Subgroup 1 (62 children) and subgroup 2 (50 children) were different for fixation (*p* = 0.02), visual acuity (*p* = 0.03) and contrast sensitivity (*p* < 0.01), being more frequently impaired in younger children. Comparing subgroup 2 with subgroup 3 (68 children), the older children presented more frequently myopia (*p* = 0.02) while the younger ones esotropia (*p* = 0.02) and alteration in smooth pursuit (*p* = 0.03) and saccades (*p* < 0.01). Furthermore, fixation, smooth pursuit, visual acuity, contrast sensitivity and visual filed (*p* < 0.01) were more frequently impaired in younger children (subgroup 1) compared to the older ones. Multiple correspondence analysis (MCA) confirmed the different neurovisual profiles according to age: younger children with CP showed more signs of CVI compared to the older ones. 34 out of 68 children belonging to subgroup 3 underwent the cognitive visual evaluation; an impairment of cognitive visual skills was detected in 21 subjects.

**Conclusion:**

Younger children with CP showed more signs of CVI compared to the older ones, likely for the physiological maturation of visual system and mechanisms of neuroplasticity. In this direction, we suggest an early neurovisual evaluation to detect any weak visual functions.

## Introduction

Cerebral Visual Impairment (CVI) is the major non-ocular cause of pediatric visual impairment worldwide (Bauer and Merabet, 2019; Ortibus et al., 2019; Philip et al., 2020; Tinelli et al., 2020; Sakki et al., 2021) and it is operationally defined as “a verifiable visual dysfunction, which cannot be attributed to disorders of the anterior visual pathways or any potentially co-occurring ocular impairment” (Sakki et al., 2018; Bauer and Merabet, 2019). In recent years, there has been an increased effort to find a consensual definition of CVI (Kran et al., 2019; Ortibus et al., 2019; Sakki et al., 2021), and to identify a classification system based on clinical severity (Philip and Dutton, 2014; Sakki et al., 2021). Sakki et al. (2021) used cluster analysis to derive a medically based CVI classification, identifying three different profiles: (A1) selective visual perception and visuomotor deficits; (A2) more severe and broader visual perception and visuomotor deficits, and variable visual acuity; (B) unable to perform psychological testing (significant visual acuity reduction). The three subgroups showed profiles progressively more severe from Group A1 to Group B also for refractive errors, strabismus, nystagmus and the level of motor impairment with the majority of children belonging to Group B with Cerebral Palsy (CP). It is widely known that CVI is frequently observed in CP (Fazzi et al., 2007). We have found a great variety in its prevalence among studies (from 16 to 70%) according to the sources of clinical information used (for example direct observation, telephone questionnaires), to the different definition of visual impairment, to the methodological heterogeneity (for example different tests used for assessing the cognitive visual disorders), and to the visual parameters taken at clinical assessment (Ego et al., 2015; Duke et al., 2020; Philip et al., 2020; Tinelli et al., 2020).

The co-occurrence of CVI and CP is related to the fact that the lesions to motor pathways, particularly periventricular leukomalacia, are anatomically close to visual pathways (Fazzi et al., 2012; Tinelli et al., 2020).

The clinical spectrum of visual problems in children affected by CP is extremely broad, ranging from mild to severe, and including ophthalmological, oculomotor, basic visual function, cognitive-visual disorders (CVDs) (Fazzi et al., 2007; Castelli and Fazzi, 2016; Merabet et al., 2016; Maioli et al., 2019; Baranello et al., 2020; Bennett et al., 2020). In children affected by CP, the severity of visual impairment seems to correlate with the severity of motor deficits (Dufresne et al., 2014). Fazzi et al. (2012) describe different neuro-ophthalmological profiles according to the type of CP. Children with tetraplegic CP showed the greatest visual impairment, presenting markedly reduced or not assessable visual acuity, highly impaired or absent oculomotor functions, and high percentage of ocular abnormalities. Diplegic CP was characterized by moderately reduced visual acuity, altered contrast sensitivity, absent stereopsis, impaired oculomotor abilities, refractive errors and CVDs while children suffered from hemiplegic CP presented slight reduced visual acuity, reduced visual field (frequently unilateral), altered stereopsis, less frequent oculomotor involvement, and refractive errors. Tinelli et al. (2020) also suggest a relationship between brain lesion severity and visual function in children affected by CP: visual acuity, visual field, stereopsis and color perception were compromised in presence of a cortical damage, while oculomotor functions in presence of a subcortical damage. Therefore, to date authors have focused on profiling the spectrum of CVI according to the type of CP (Fazzi et al., 2012) or to etiology, location, timing and extent of brain lesions (Guzzetta et al., 2001; Bennett et al., 2020; Tinelli et al., 2020).

Although visual functions progressively mature during the first years of life in healthy subjects (Luna et al., 2008; Lewis and Maurer, 2009; Helo et al., 2014), only few studies evaluated the visual profile in children affected by CP according to age, reporting inconsistent results. Ego et al. (2015) hypothesized a spontaneous improvement of oculomotor functions, quantitatively assessed, in a cohort of children with CP, while Tinelli et al. (2020) found no correlation between the age of CP subjects and the Visual Total Score (Tinelli et al., 2020) obtained from the sum of oculomotor (fixation, following, saccades, and nystagmus) and perceptual signs (acuity, binocular visual fields, stereopsis and color perception); for each item, the authors gave a score of 0 if “not compromised” or of 1 “when there is an impairment.” However, the visual dysfunctions considered in Visual Total Score can occur independently and their sum may hide potential underlying associations. Finally, literature data on visual function outcome in children with CVI caused by heterogeneous etiology (such as cerebral nervous system malformations, infections, injuries or seizures) reported an improvement of visual acuity, ranging between 32–83% (Matsuba and Jan, 2006; Handa et al., 2018) and contrast sensitivity (Watson et al., 2007).

Profiling the visual development of patients affected by CVI and CP is a crucial starting point to ameliorate their follow-up. Understanding the developmental trajectories of each impaired visual function may allow health professionals to: (1) define the type and the timing of rehabilitation, directing resources toward those functions that have till the possibility for improvement and, at the same time, preventing them from being further compromised; (2) advance the awareness and understanding of mild spectrum of CVI that can go unrecognized until it interferes with learning and daily life activities; (3) improve counseling offered to families regarding the developmental trajectories of each impaired visual function.

Our hypothesis is that the clinical spectrum of CVI can modify during the first years of life and that the youngest children can present more signs of CVI in terms of visual dysfunctions compared to the older ones. In fact, in literature we found data that attests that age-related visual development is due to maturation of brain anatomy and function (Luna et al., 2008). Moreover, visual deficits caused by early brain damage could be influenced by adaptive neuroplasticity that, especially during the first years of life, modulate the natural history of children suffering from CP and CVI (Ismail et al., 2017; Sabel et al., 2018; Fiori et al., 2019).

The aims of our study were: (1) to detail the neurovisual profile (that means ophthalmological, oculomotor and basic visual functions) of a large group of children affected by CP according to three different age subgroups (subgroup 1: infants 6 months–2 years; subgroup 2: pre-school age 3–5 years; subgroup 3: school age > 6 years), (2) to compare age subgroups. Finally, (3) we wanted to know whether cognitive visual functions in the oldest age group were different from reference values and whether the presence of cognitive visual disorder was related to the IQ values (FIQ, VIQ and PIQ). We divided the sample into these three age subgroups based on visual anatomical and functional aspects. Gross anatomical structures, although constructed before birth, continue to develop into childhood, together with the maturation of neural circuits of the visual cortex (Kovács et al., 1999). Specifically, some authors suggest the hypothesis that synaptogenesis in human visual cortex reaches a peak between 8 months and 2 years and is followed by a long period of synaptic pruning to reach adult levels later in childhood (Siu and Murphy, 2018). A similar trajectory is seen in dendritic refinement, with a peak at 5 months and adult levels by 2 years (Siu and Murphy, 2018). Many anatomical features (as cortical thickness, synaptogenesis, horizontal, and interlaminar connections, for details see the review of Siu and Murphy (2018)) are already adult-like at this stage, but vision continues to mature well beyond the first years of life. In fact, lower visual functions (such as visual acuity) reach adult level between 3 and 5 years of age, while higher level visual function (such as figure-ground discrimination and visual attention) complete their development in adolescence (Dutton, 2003; Ortibus et al., 2019). However, the precise age of maturation may vary significantly depend on test design (e.g., because of potential validity issues), that in turn hinders estimations of function development and on the visual experience of the child. Finally, literature data report that the time windows of (critical) visual function development, which could increase the opportunity for effective treatment, are under 5 years of age (Siu and Murphy, 2018).

## Materials and Methods

### Participants

193 children with CP were referred to our Neuro-ophthalmological Tertiary Center of Child Neurology and Psychiatry Unit, Civil Hospital of Brescia, between July 2017 and July 2020, by medical specialists, as pediatricians and child neurologists and psychiatrists, because of a visual impairment screening. Of these, 13 children (infants 6 months–2 years: 5 cases; pre-school age 3–5 years: 3 cases; school age > 6 years: 5 cases) were excluded from the study because they did not show any visual signs. The remaining 180 (104 males, 76 females) met the inclusion criteria and were selected for this study. Inclusion criteria were: diagnosis of CP, confirmed by neurological examination and brain Magnetic Resonance Imaging (MRI); age between 6 months and 18 years; presence of CVI according to Sakki et al. (2018, 2021). We made a CVI diagnosis according to the European definition “a verifiable visual dysfunction, which cannot be attributed to disorders of the anterior visual pathways or any potentially co-occurring ocular impairment.” We enrolled all the CP subjects with a variable association of: oculomotor dysfunctions (abnormalities in fixation and/or smooth pursuit and/or saccades and/or abnormal ocular movements); basic visual function deficits (reduced visual acuity and/or visual field and/or altered contrast sensitivity); CVDs and larger optic disc cupping associated with optic nerve hypoplasia due to mechanism of trans-synaptic degeneration (Jacobson et al., 1997). These visual signs were not primarily caused by disorders of the anterior visual pathways (globe, retina, or anterior optic nerve).

The sample did not include any children presenting severe visual deficits due to abnormalities of the anterior segment or sequelae of retinopathy of prematurity in order to exclude subjects affected by mainly ocular visual impairment.

The study was conducted in accordance with the ethical guidelines established by the Declaration of Helsinki and was approved by the Ethics Committee of Brescia (NP 3070). Written informed consent was obtained by all participants and/or their parents before data collection.

### Procedure

A demographic and clinical data collection, a neurological examination with gross and fine motor evaluation, a developmental or cognitive assessment and a video-recorded visual function examination was carried out in the children affected by CP and CVI. Data on neuroradiological findings (Conventional Brain MRI) of all CP children were also collected.

We classified CP based on criteria outlined in the Surveillance of CP in Europe algorithm Surveillance of Cerebral Palsy in Europe (2000), into four subtypes: Spastic bilateral CP, Spastic unilateral CP, Dyskinetic CP, Ataxic CP. The Gross and Fine motor function was assessed using the Gross Motor Function Classification System (GMFCS) (Palisano et al., 1997) and the Manual Ability Classification System (MACS) (Eliasson et al., 2006), respectively. To assess developmental or cognitive skills, the Griffiths Mental Developmental Scales-III (Green et al., 2017), Wechsler Preschool and Primary Scale of Intelligence III edition (WPPSI-III) (Wechsler, 2002) or the Wechsler Scales of Intelligence for Children IV edition (WISC-IV) (Wechsler, 2003) were used according to the age of the children. The developmental/intelligence quotients (IQ) were measured in standard scores and defined normal (≥ 85 standard score), mildly/moderately impaired (<85 standard score).

We carried out the visual assessment according to Fazzi and colleagues (Fazzi et al., 2012; Iodice et al., 2018) and included the evaluation of: (1) ophthalmological characteristics, detecting possible refractive errors (assessed in cycloplegia), anterior segment and ocular fundus abnormalities; dynamic retinoscopy was not carried out; (2) oculomotor functions (fixation, smooth pursuit, and saccades and orthoptic evaluation to detect strabismus, ocular motility deficit and abnormal eye movements); (3) basic visual functions (visual acuity, contrast sensitivity, visual field); (4) cognitive-visual profile, carried out in children at school-age (subgroup 3) with normal IQ or mild cognitive impairment (full-scale IQ > 50 and verbal IQ > 70 standard scores) and visual acuity not less than three-tenths in binocular vision. As regards oculomotor functions, we defined fixation as present (stable for more than 3 s) or impaired (unstable or absent); we defined smooth pursuit as present (continuous) or impaired (discontinuous or difficult to elicit/absent); we defined saccadic eye movements as present (both latency and amplitude of saccade were normal) or impaired (dysmetric and/or with increased latency or absent). Visual acuity was evaluated under maximum refractive correction with test suitable for patient’s age and cooperation using Teller Acuity Cards (Teller et al., 1986), Lea Symbols or letter optotypes (Hyvärinen et al., 1980): children belonging to subgroup 1 were evaluated using Teller Acuity Cards while children belonging to subgroup 2 and 3 using Teller Acuity Card, Lea Symbols or letter optotypes. We defined visual acuity score as normal or reduced: for children belonging to subgroup 1 we used normative data according to Teller acuity cards Handbook (Teller et al., 1986); for children belonging to subgroup 2 we applied age-specific norms according to the Current American Academy of Pediatrics guidelines updated in 2016 (Donahue et al., 2016) (normal visual acuity > 4 tenths for 36–47 months, > 5 tenths for 48–59 months and > 6 tenths for ≥ 60 months of age); for the children belonging to subgroup 3 we referred to the WHO International Classification of Disease-10 definition of visual impairment (World Health Organization [WHO], 2004) (normal vision > 8 tenths; deficient < 8 tenths). We evaluated contrast sensitivity using the Hiding Heidi Low Contrast “Face” Test (HH). Since Leat and Wegmann (2004) reported that most children aged between 1 and 8 years old correctly responded to the lowest contrast at the HH, we considered the ability to identify targets as “normal” at 1.25% contrast level and as “altered” when ≥ 2.5%. We evaluated the ability to locate targets presented in different areas of the visual field binocularly using kinetic perimetry (van Hof-van Duin et al., 1992), based on child’s behavioral reactions (e.g., movements of the head, eyes, or a limb toward the target) and we classified it as normal or reduced, according to age-specific normative data reported in the literature (Heersma et al., 1989; Wilson et al., 1991; van Hof-van Duin et al., 1992); we considered normal a result within 2 standard deviation.

We performed the cognitive-visual assessment using a battery of tests referring to visual motor and visual perceptual skills. Visual motor skills were analyzed using the Developmental Test of Visual-Motor Integration -VMI- (Beery and Buktenica, 2000), a paper-and-pencil test for visual motor integration abilities, and the Block Construction -BC- task, a subtest of NEPSY battery (Korkman et al., 2011), for constructional praxia. Visual perceptual skills, in children < 11 years old, were assessed using the Bova et al. (2007) battery that includes the evaluation of: (1) The perceptual categorization, that means the ability to recognize the structural identity of an object when its projection on the retina is altered (using the Street Completion Test (Street, 1931) - SCT-, colored photographs of objects viewed from unusual perspectives - UP-, photographs illuminated in unusual ways -UI-), (2) The constancy of internal representation of objects (using a series of Imaginary Figures -IF-), and (3) The Semantic categorization, which is the capacity of recognizing semantic and functional attributes of stimuli (using Matching Tasks respectively -MC- and -MF-). We used the Street Completion Test on children > 11 years old to evaluate visual perceptual skills. Visual motor and visual perceptual functions were considered impaired if z score derived from normal controls was under -2 on at least one of the tasks evaluated. A CVD was considered present in case of visual motor and/or visual perceptual dysfunction.

A multidisciplinary team carried out the visual function evaluation: a child neuropsychiatrist performed the oculomotor/basic visual functions assessment supported by a child therapist who conducted the video-recording; an ophthalmologist performed the ophthalmology evaluation, an orthoptist detected the presence of strabismus, ocular motility deficit and abnormal eye movements and a neuropsychologist assessed the cognitive visual functions. The video-recorded examination allows the teams to observe and judge the child performance (especially the qualitative functions as fixation, smooth pursuit and saccadic movements).

We classified Brain MRIs according to the MRI Classification System proposed by the Surveillance of Cerebral Palsy in Europe (Himmelmann et al., 2017), that consists of five main groups: (A) maldevelopments, (B) predominant white matter injury, (C) predominant gray matter injury, (D) miscellaneous, and (E) normal finding.

### Statistical Analysis

Demographic data, clinical features (subtype of cerebral palsy, level of gross and fine motor impairment, IQ) and the neuroimaging findings of the entire sample and of the three subgroups were described using means, standard deviation, and range for quantitative variables (gestational age, birth weight) and counts and percentage for qualitative variables (subtype of cerebral palsy, level of gross and fine motor impairment, IQ and brain MRI classification). Comparison between age subgroups and these variables were performed using Kruskal-Wallis test for quantitative variables and Chi squared test for qualitative variables.

For the first aim, data on neurovisual profile according to the three different age subgroups were described using means, standard deviation, and range for quantitative variables (visual acuity and contrast sensitivity) and counts and percentage of impaired qualitative variables (ophthalmological, oculomotor functions and basic visual functions). For the second aim, we compared the evolution of neurovisual profiles between the different age subgroups using a logistic regression model for all visual variables (ophthalmological, oculomotor and basic visual functions), results were reported as odds ratio (OR) and 95% Confidence Interval (CI). We carried out a multiple correspondence analysis (MCA) to investigate the relationships between categorical variables: refractive errors, fundus oculi abnormalities, strabismus, nystagmus, fixation, smooth pursuit and saccadic alterations, abnormal visual acuity, altered contrast sensitivity and visual field deficit (visual motor and visual perceptual disorders were not included in the analysis because assessed only in the subgroup 3). The 10 visual items were dichotomized (“yes/no” when the visual disorder was present/absent). The MCA approach provides coordinate plots that can be graphically interpreted as follow: (1) variable categories with a similar profile are grouped together; (2) negatively correlated variable categories are positioned on opposite sides of the plot origin (opposed quadrants); (3) the distance between category points and the origin measures the quality of the variable category on the factor map; (4) category points that are away from the origin are well represented on the factor map. The quality of the representation of each variable is called the squared cosine (cos2), which measures the degree of association between variable categories and a particular axis. We added age (categorized) as a supplementary variable, that is, it was not used to generate the principal dimensions but rather the category coordinates were projected on the coordinate plot defined by the active variables (10 visual items). Multiple comparisons’ *p*-values were adjusted using Tukey algorithm.

For the third aim, data on cognitive visual functions in the oldest age group were described using counts and percentage of the impaired visual motor and visual perceptual variables. The relationship between the presence of cognitive visual disorder and IQ values (FIQ, VIQ, and PIQ) was evaluated using logistic regression model; results were reported as OR and 95% CI.

All analyses were performed using R statistical package (version 4.0.3) assuming a significance threshold of 5%.

## Results

**FIGURE 1:**
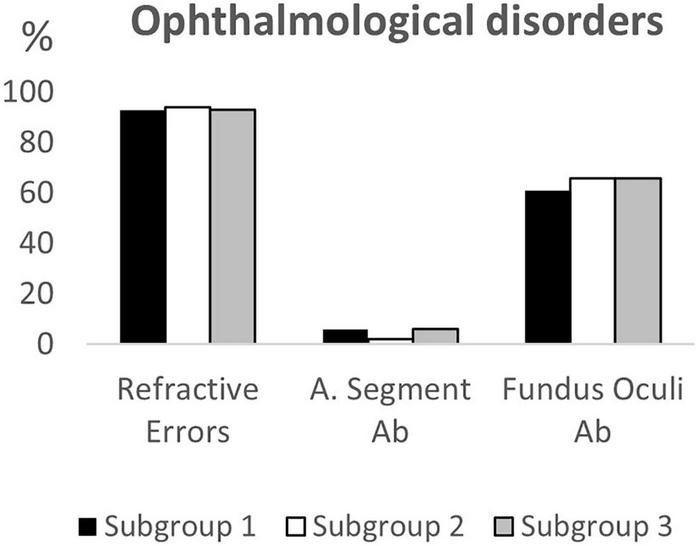
Ophthalmological disorders according to age subgroups. A. Segment Ab, Anterior Segment abnormalities; Fundus Oculi Ab, Fundus Oculi abnormalities.

**FIGURE 2:**
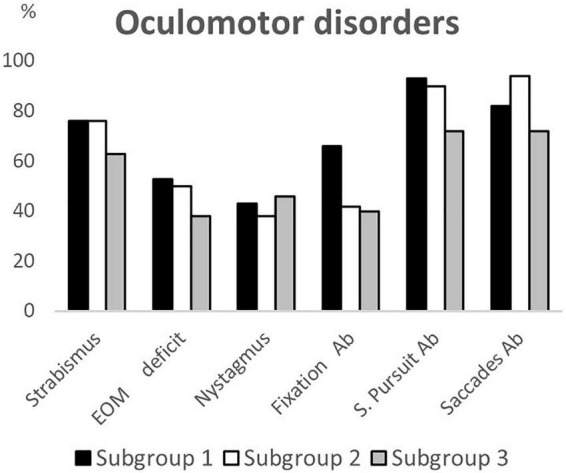
Oculomotor disorders according to age subgroups. EOM deficit, extrinsic ocular motility deficit; Ab, abnormalities; S. Pursuit, smooth pursuit.

**FIGURE 3:**
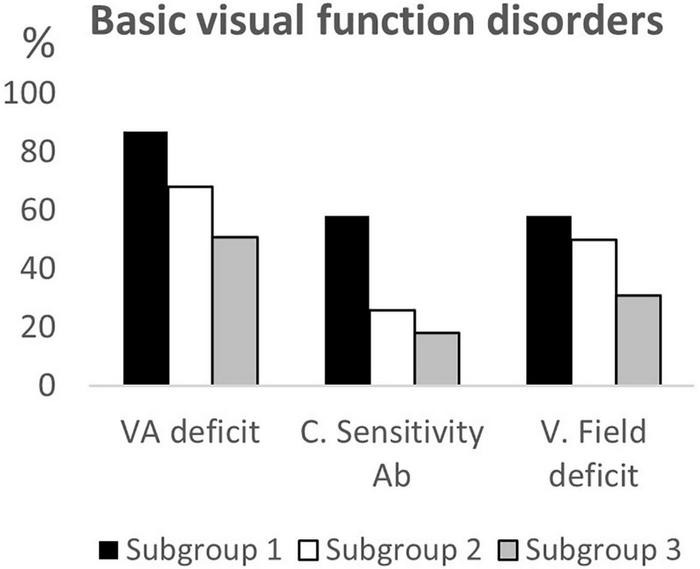
Basic visual function disorders according to age subgroups. VA deficit, visual acuity deficit; C. Sensitivity Ab, Contrast Sensitivity abnormalities; V. field deficit, visual field deficit.

Of the 180 children selected for this study, 62 belonged to subgroup 1, 50 to subgroup 2 and 68 to subgroup 3. Table 1 summarizes the demographic data, the clinical features and the neuroimaging findings of the entire sample and of the three subgroups; these characteristics were comparable between each of the three subgroups. Data on neurovisual profiles (ophthalmological, oculomotor and basic visual functions) of the three subgroups are summarized in Table 2 and Figures 1–3.

**TABLE 1:** Demographic, anamnestic, clinical, and neuroradiological characteristics of the sample and of the age subgroups.

	Total sample	Subgroup 1 (6 mo-2 yr)	Subgroup 2 (3–5 yr)	Subgroup 3 (> 6 yr)	*P*-value
**N subjects**	180	62	50	68	
**Mean age (mo) ± SD (range)**	66 ± 42 (6–192)	21 ± 8.3 (6–35)	58 ± 9.9 (36–71)	111 ± 25.8 (75–192)	
**Male/Female distribution N (%)**	104(58)/76(42)	37(60)/25(40)	31(62)/19(38)	36(53)/32(47)	0.576
**Mean GA (wks) ± SD (range)**	34.9 ± 5 (24–42)	35 ± 5 (24–41)	35 ± 5.2 (25–41)	34.7 ± 5 (24–42)	0.838
**Preterm birth N (%)**	105(59.0)	33(53.2)	28(57.1)	44(65.7)	0.280
**Mean birth weight (g) ± SD (range)**	2289 ± 968 (380–4860)	2265 ± 936 (620–3700)	2343 ± 1,068 (380–4630)	2270 ± 932 (800–4860)	0.935
**Type of cerebral palsy:**					0.722
Spastic unilateral, N (%)	63 (35)	17 (27)	18 (36)	29 (43)	
Spastic bilateral, N (%)	94 (52)	37 (60)	27 (54)	29 (43)	
Dyskinetic, N (%)	20 (11)	8 (13)	4 (8)	8 (12)	
Ataxic, N (%)	3 (2)	0	1 (2)	2 (3)	
**Gross motor involvement:**					0.613
Mild (GMFCS level 1 or 2), N (%)	90 (50)	28 (45)	25 (50)	37 (54)	
Moderate (GMFCS level 3), N (%)	7 (4)	2 (3)	1 (2)	4 (6)	
Severe (GMFCS level 4 or 5), N (%)	83 (46)	32 (52)	24 (48)	27 (40)	
**Fine motor involvement:**					0.769
Mild (MACS level 1 or 2), N (%)	102 (57)	32 (52)	29 (58)	41 (60)	
Moderate (MACS level 3), N (%)	25 (14)	9 (14)	7 (14)	9 (13)	
Severe (MACS level 4 or 5), N (%)	53 (29)	21 (34)	14 (28)	18 (27)	
**Developmental/Cognitive quotient:**					0.623
Normal, N (%)	55 (31)	17 (27)	18 (36)	20 (29)	
Mild/moderate impaired, N (%)	125 (69)	45 (73)	32 (64)	48 (71)	
**Brain MRICS:**					0.649
MRICS type A, N (%)	7 (4)	2 (3)	3 (6)	2 (3)	
MRICS type B, N (%)	106 (59)	38 (61)	28 (56)	40 (59)	
MRICS type C, N (%)	49 (27)	18 (29)	11 (22)	20 (29)	
MRICS type D, N (%)	18 (10)	4 (7)	8 (16)	6 (9)	

*N, number; mo, months; yr, years; SD, Standard deviation; GA, gestational age; wks, weeks; g, grams; GMFCS, gross motor function classification system; MACS, manual ability classification system; MRICS, magnetic resonance imaging classification system.*

**TABLE 2:** Neurovisual profiles between the three subgroups.

	Subgroup 1 (*N* = 62)	Subgroup 2 (*N* = 50)	Subgroup 3 (*N* = 68)	Comparison between subgroups, OR (CI 95%); *p*-value			
				1 vs. 2	2 vs. 3	1 vs. 3	
**Refractive errors N**	**58**	**47**	**63**	*1.08 (0.1; 6.2*); *p* = *0.98*	0.80 (0.1; 4.0); *p* = *0.93*)	0.87 (0.1; 4.0); *p* = *0.96*	1 = 2 = 3
Mixed refractive errrors	42	35	44	1.1 (0.4; 2.7); *p* = *0.94*	0.7 (0.3; 1.8); *p* = *0.76*	0.8 (0.3; 1.9); *p* = *0.89*	1 = 2 = 3
Astigmatism (isolated/mixed)	52	41	54	0.88 (0.2; 2.6); *p* = *0.93*	0.85 (0.3; 2.4); *p* = *0.91*	0.74 (0.2; 2.0); *p* = *0.72*	1 = 2 = 3
Hypermetropia (isolated/mixed)	37	33	31	1.31 (0.5; 3.1); *p* = *0.70*	0.43 (0.1; 1.0); ***p* = *0.052***	0.57 (0.2; 1.2); *p* = *0.19*	1 = 2 > 3
Myopia (isolated/mixed)	13	8	25	0.72 (0.2; 2.1); *p* = *0.71*	3.05 (1.1; 8.3); ***p* = *0.02***	2.19 (0.9; 5.3); *p* = *0.09*	1 = 2 < 3
**Anterior segment Ab N**	**4**	**1**	**4**	0.3 (0.02; 3.6); *p* = *0.45*	3.06 (0.2; 35.2); *p* = *0.44*	0.9 (0.1; 4.5); *p* = *0.98*	1 = 2 = 3
**Fundus oculi Ab N**	**38**	**30**	**45**	0.95 (0.4; 2.2); *p* = *0.97*	1.30 (0.5; 3.0); *p* = *0.70*	1.24 (0.5; 2.7); *p* = *0.77*	1 = 2 = 3
Disc pallor	18	16	27	1.15 (0.4; 2.8); *p* = *0.9*	1.4 (0.5; 3.3); *p* = *0.58*	1.61 (0.7; 3.6); *p* = *0.34*	1 = 2 = 3
Disc cupping	9	3	6	0.38 (0.08; 1.7); *p* = *0.27*	1.52 (0.3; 7.5); *p* = *0.76*	0.57 (0.1; 1.9); *p* = *0.5*	1 = 2 = 3
Disc pallor, cupping, nerve hy	11	11	12	1.31 (0.4; 3.7); *p* = *0.78*	0.76 (0.2; 2.1); *p* = *0.77*	0.99 (0.3; 2.7); *p* = *1.00*	1 = 2 = 3
**Strabismus N**	**47**	**38**	**43**	1.01 (0.3; 2.7); *p* = *0.99*	0.54 (0.2; 1.3); *p* = *0.23*	0.55 (0.2; 1.3); *p* = *0.21*	1 = 2 = 3
Esotropia	30	26	20	1.16 (0.5; 2.6); *p* = *0.88*	0.38 (0.1; 0.9)*;* ***p* = *0.02***	0.44 (0.2; 1.0); ***p* = *0.052***	1 = 2 > 3
Exotropia	17	12	23	0.86 (0.3; 2.2); *p* = *0.89*	1.45 (0.5; 3.6); *p* = *0.54*	1.25 (0.5; 2.9); *p* = *0.77*	1 = 2 = 3
**EOM deficit N**	**33**	**25**	**26**	0.88(0.3; 2.0); *p* = *0.9*	0.62 (0.2; 1.4); *p* = *0.33*	0.54 (0.2; 1.2); *p* = 0.15	1 = 2 = 3
Abduction deficit	25	19	20	0.91 (0.3; 2.1); *p* = *0.94*	0.68 (0.2; 1.6); *p* = *0.5*	0.62 (0.2; 1.4); *p* = *0.32*	1 = 2 = 3
Upshoot in adduction	3	2	3	0.82 (0.1; 6.5*); p* = *0.95*	1.1 (0.1; 8.6); *p* = *0.99*	0.91 (0.1; 5.8); *p* = *0.98*	1 = 2 = 3
Upshoot in abduction	5	4	3	0.99(0.2; 4.6); *p* = *1.00*	0.53 (0.09; 3.0); *p* = *0.63*	0.53 (0.1; 2.8); *p* = *0.59*	1 = 2 = 3
**Nystagmus N**	**27**	**19**	**31**	0.79 (0.3; 1.8); *p* = *0.76*	1.37 (0.5; 3.1); *p* = *0.60*	1.09 (0.5; 2.3); *p* = *0.95*	1 = 2 = 3
**Fixation Ab N**	**41**	**21**	**27**	0.37 (0.1; 0.8); ***p* = *0.02***	0.91 (0.3; 2.1); *p* = *0.95*	0.34 (0.1; 0.7); ***p* < *0.01***	1 > 2 = 3
**Smooth pursuit Ab N**	**58**	**45**	**49**	0.62 (0.1; 2.9); *p* = *0.71*	0.2 (0.09; 0.9); ***p* = *0.03***	0.1 (0.05; 0.6); ***p* < *0.01***	1 = 2 > 3
**Saccadic Ab N**	**51**	**47**	**49**	3.38 (0.7; 15.3); *p* = *0.13*	0.16 (0.04; 0.6); ***p* < *0.01***	0.56 (0.2; 1.4); *p* = *0.29*	1 = 2 > 3
**Visual acuity deficit**	**54**	**34**	**35**	-1.1 (-2.2; -0.08); ***p* = *0.03***	-0.69 (-1.5; 0.1); *p* = *0.13*	-1.85 (-2.8; -0.8); ***p* < *0.01***	1 > 2 = 3
N of sbj: Teller Acuity Cards Mean value in cy/cm ± SD Range	62 3.4 ± 2.5 0.23–19	20 3.9 ± 4.1 0.32–13	14 3.1 ± 3.3 0.43–13				
N of sbj: Lea Sy; letter O Mean value in tenths ± SD Range	/	30: 22; 8 5.9 ± 2.9 1–10	54: 4; 50 7.2 ± 3 1–10				
**Contrast sensitivity Ab N**	**36**	**13**	**12**	0.25 (0.1; 0.6); ***p* < *0.01***	0.61 (0.2; 1.6); *p* = *0.43*	0.1 (0.06; 0.3); ***p* < *0.01***	1 > 2 = 3
Mean value in % ± SD	38.6 ± 44.7	23.6 ± 41.1	8.8 ± 23.9				
Range in %	1.25–100	1.25–100	1.25–100				
**Visual field deficit N**	**36**	**25**	**21**	0.72 (0.3; 1.6); *p* = *0.6*	0.45 (0.1; 1.0); *p* = *0.06*	0.32 (0.1; 0.7); ***p* < *0.01***	1 = 2 > 3
Right or left field defect	16	12	12	0.91 (0.3; 2.4); *p* = *0.95*	0.68 (0.2; 1.8); *p* = *0.6*	0.62 (0.2; 1.6); *p* = *0.42*	1 = 2 = 3
Upper or inferior field defect	2	1	1	0.61 (0.04; 9.6); *p* = *0.87*	0.7 (0.03; 16.9); *p* = *0.96*	0.45 (0.03; 7); *p* = *0.73*	1 = 2 = 3
Generalized field loss	18	12	8	0.77 (0.2; 2.0); *p* = *0.76*	0.42 (0.1; 1.2); *p* = *0.15*	0.33 (0.1; 0.9); ***p* = *0.03***	1 = 2 > 3

*N, number; Ab, abnormalities; hy, hypoplasia; EOM, extrinsic ocular motility; Lea Sy, Lea symbols, letter O, letter optotype. Bold and italic values represent significant findings.*

### Comparison of the Neurovisual Profiles Between the Three Subgroups

Comparing the neurovisual profiles between subgroup 1 and subgroup 2, we observed that CVI signs (refractive errors, fundus oculi abnormalities, strabismus, ocular motility deficits, nystagmus, altered smooth pursuit and saccades, and visual field deficits) did not significantly differ, except for fixation (*p* = 0.02), visual acuity (*p* = 0.03) and contrast sensitivity (*p* < 0.01) that were more frequently impaired in younger children (subgroup 1). From the comparison of the neurovisual profiles between subgroup 2 and subgroup 3, we found no differences in refractive errors (although myopia was more frequent, *p* = 0.02, and hypermetropia less frequent, *p* = 0.052 in the subgroup 3), fundus oculi abnormalities and nystagmus. The two subgroups significantly differed for esotropia (*p* = 0.02), and alteration of smooth pursuit (*p* = 0.03) and saccades (*p* < 0.01), more frequently present in subgroup 2.

**FIGURE 4:**
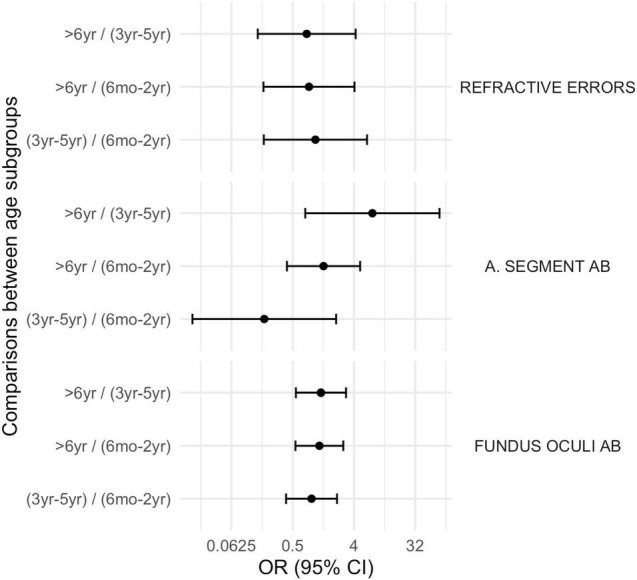
Comparison between subgroups, OR (CI 95%): ophthalmological disorders. A. Segment Ab, Anterior Segment abnormalities; Fundus Oculi Ab, Fundus Oculi abnormalities.

**FIGURE 5:**
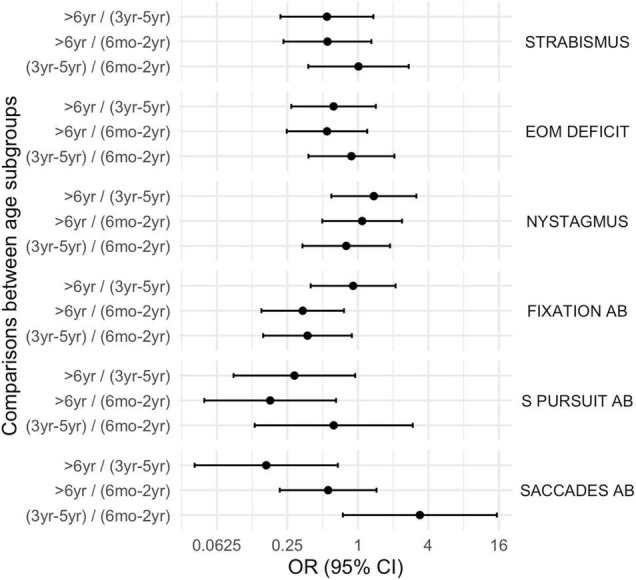
Comparison between subgroups, OR (CI 95%): oculomotor disorders. EOM deficit, extrinsic ocular motility deficit, Ab, abnormalities, S. Pursuit, smooth pursuit.

**FIGURE 6:**
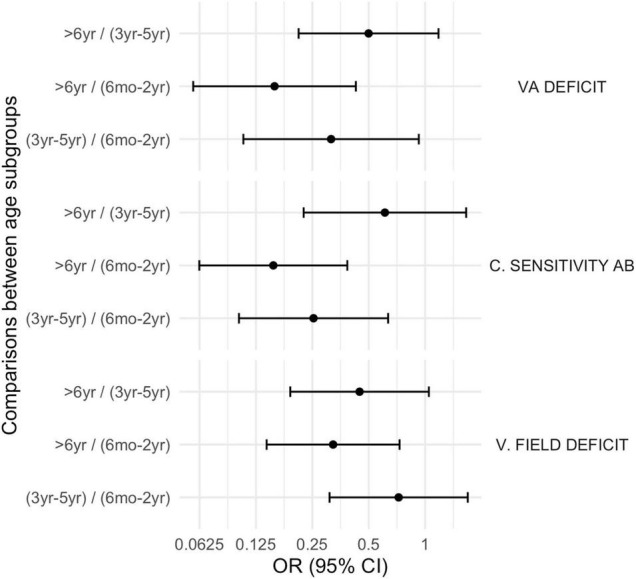
Comparison between subgroups, OR (CI 95%): basic visual function disorders. VA deficit, visual acuity deficit; C. Sensitivity Ab, Contrast Sensitivity abnormalities; V. field deficit, visual field deficit.

Moreover, neurovisual profile was significantly different between subgroup 1 and 3 for fixation, smooth pursuit, visual acuity, contrast sensitivity and visual filed (*p* < 0.01) being less frequently impaired in the older children (subgroup 3). Findings are summarized in Table 2 and in Figures 4–6.

### MAC Analysis

**FIGURE 7:**
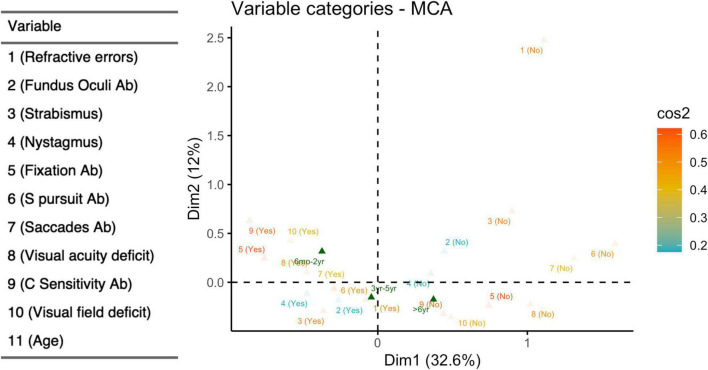
Multiple correspondence analyses (dichotomized items). Coordinate plot: MCA of 10 visual items after dichotomization; supplementary variables: age subgroup, defined as 6 months-2 years, 3–5 years, > 6 years. Explained variability: 1st dimension (horizontal axis) 32.6%; 2nd dimension (vertical axis) 11.9%. Yes (presence of visual deficit); No (absence of visual deficit); Ab (abnormalities).

**FIGURE 8:**
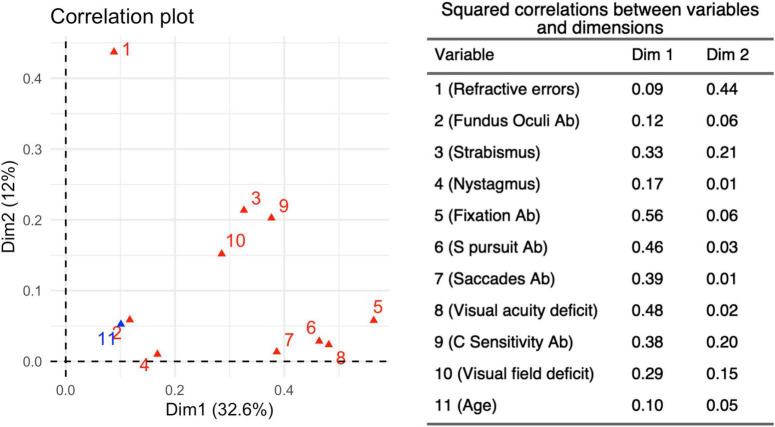
Squared Correlations (r^2^) between each variable and the first two main Dimensions 1 and 2. The *r*^2^-values related to the two dimensions determined by the MCA for 10 variables, identified from the neurovisual evaluation carried out in 180 children belonging to the three different age subgroups. Dimension 1 accounts for 32.6% of the variance in this analysis, while Dimension 2 accounts for 12%.

MCA (Figure 7) was able to explain 46% of the total variation using the two main dimensions (Supplementary Figure 1). The coordinate plot documented two possible average profiles. In the first, the 10 visual items were predominantly impaired, while the second was characterized by the absence of visual problems. We added age subgroups as a supplementary variable in order to address the interpretation of those profiles: the younger children (6 months – 2 years) fitted perfectly in the first profile, while the older ones (> 6 years) were consistent with the second profile. Only the presence/absence of refractive errors remained distant from the two profiles, probably because they were extremely frequent in all three age-subgroups and they seem basically unrelated to visual dysfunctions. The squared correlation of each variable to the first two MCA dimensions are provided in Figure 8.

### Cognitive-Visual Evaluation in Subgroup 3 (>6 Years)

Thirty-four out of 68 children (50%) met the criteria for the cognitive-visual evaluation. The mean age was 112.9 months (SD 28, range 72–192 months), 17 (50%) were females and 17 males (50%). The mean gestational age was 35.5 weeks (SD 5.2, range 24–42 weeks), 14 (41%) were born preterm and 20 (59%) at term; the mean birth weight was 2,408 g (SD 921.1, range 860–3,810 g). 20 children (59%) presented a spastic unilateral CP and 14 (41%) a spastic bilateral CP; the level of gross motor impairment at GMFCS evaluation was mild in 27 cases (79%), moderate in 2 (6%) and severe in 5 subjects (15%), while the level of manual ability impairment at MACS was mild in 29 children (85%) and moderate in 5 (15%). The mean full IQ was 82.1 standard scores -s.s.- (SD 17.2, range 50–114 s.s.), the mean Verbal IQ was 93.2 s.s. (SD 14.9, range 70–139 s.s.) and the mean Performance IQ was 77.5 s.s. (SD 22, range 43–127 s.s.). Brain MRIs lesion were classified as predominant white matter injury in 22 subjects (65%), predominant gray matter injury in 10 (29%) and miscellaneous in 2 cases (6%).

**FIGURE 9:**
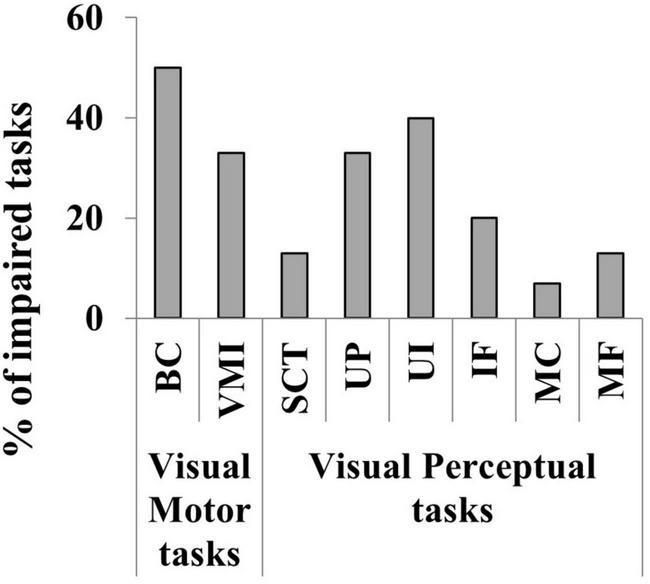
Percentage of the impaired cognitive visual tasks in the 34 children evaluated. Visual motor skills (BC, Block Construction task; VMI, Developmental Test of Visual-Motor Integration) and Visual perceptual skills (STC, Street Completion Test; UP, colored photographs of objects viewed from unusual perspectives; UL, photographs illuminated in unusual ways; IF, Imagery Figures; MC, Matching Tasks for the ability to recognize semantic attributes of stimuli; MF, Matching Tasks for the ability to recognize functional attributes of stimuli).

30 children aged < 11 years underwent all the 8 tasks for cognitive visual assessment while 4 aged > 11 years completed the VMI, BC and SCT. An impairment of cognitive visual skills was detected in 21 out 34 subjects (18 aged < 11 years; 62%). See Table 3 for details on impaired cognitive visual performances of the 21 children. BC, VMI, UP and UL seemed to be the most frequently impaired tasks as detailed in Figure 9. The statistical analysis performed to evaluate the impact of IQ on cognitive visual disorders, revealed positive associations between FIQ and CVDs (OR 0.92; CI 95%: 0.85, 0.97; *p* = 0.008), PIQ and CVDs (OR 0.91; CI 95%: 0.83, 0.96; *p* = 0.006); no associations emerged between VIQ and CVDs (OR 0.97; CI 95%: 0.92, 1.02; *p* = 0.2).

**TABLE 3:** Details on cognitive visual tasks of children with a CVD (belonging to subgroup 3).

Sbj	Age (yr, mo)	FIQ/VIQ/PIQ	VA	Cognitive-visual disorders (z-scores)							
				Visual motor tasks		Visual perceptual tasks					
				BC	VMI	SCT	UP	UL	IF	MC	MF
1	6	114/139/77	0.7	–2	–3.2	–1.3	–1.4	–4.9	0	–9.6	–6.6
2	6,7	78/114/50	0.5	–1.6	–3.1	–1.3	–1.7	–2.5	–0.7	–3.6	–0.6
3	7,2	73/97/45	0.4	–3	–4	–1.9	–3.2	–2.1	–1.4	–1.6	–1.4
4	7,3	87/90/87	0.5	–2.8	–2	0.4	–0.5	–0.8	0.8	0.3	0.8
5	7,4	70/88/43	0.5	–8.1	–2.7	–3.6	–2.2	–2	–1	0	0
6	7,5	76/81/76	0.9	–2.3	–1.5	0.6	–0.5	0.6	–0.6	0	0
7	7,6	53/88/63	0.3	–6.4	–6.4	–2.7	–4.6	–7.3	–5.2	0	0.7
8	8,1	85/92/82	1	–2	–1.6	0.6	–0.2	1.1	0.5	0	0.7
9	8,1	55/70/48	0.8	–2.6	–2.2	–0.4	–1.4	–3.9	0.4	0	–2.8
10	8,5	87/99/77	0.9	–3	–2	–2.3	–2.5	–0.8	–1.8	0.3	0.7
11	9	99/92/107	0.6	–0.3	0.5	0.4	–1.6	–2.5	0	0	0.7
12	9	50/70/48	0.9	–2.6	–2.7	–1	–2.1	–2	–1.2	0	–2.1
13	9	77/92/66	0.9	–2	–1.9	–1.6	–2.7	–2	–3.5	0	0.4
14	9,5	75/102/52	1	–3	–3.7	0.4	–4.8	–6.2	–2.8	–1.8	–2.8
15	10	57/70/53	1	–2	–1.8	–2.3	–2.5	–5.4	–5.5	0	0
16	10,8	67/70/77	0.6	–1.3	–1.1	–0.2	–2.5	0	–2.4	0	0
17	11	70/70/83	1	–2	–1.7	0.6	–0.9	0	0.2	0	0
18	11	70/100/56	1	–2.7	–2.4	–0.8	–4.5	–3	–10.3	0	0
19	11,8	55/77/45	1	–3.3	–2.1	–2.3	/	/	/	/	/
20	13,6	97/110/93	1	–1.3	–0.8	–2.8	/	/	/	/	/
21	16	83/93/76	1	–3	–2.7	–2.1	/	/	/	/	/

*Sbj, subject; yr, years; mo, months; FIQ, full intelligence quotient; VIQ, verbal intelligence quotient; PIQ, performance intelligence quotient; VA, visual acuity in tenths; BC, Block Construction task; VMI, Developmental Test of Visual-Motor Integration; STC, Street Completion Test; UP, colored photographs of objects viewed from unusual perspectives; UL, photographs illuminated in unusual ways; IF, Imagery Figures; MC, Matching Tasks for the ability to recognize semantic attributes of stimuli; MF, Matching Tasks for the ability to recognize functional attributes of stimuli.*

## Discussion

Cerebral Visual Impairment in children with CP is researched in current literature and considered a core symptom of CP on account of its high prevalence (Dufresne et al., 2014) and its impact on daily life (Pavlova and Krägeloh-Mann, 2013). Hence, in the present study we aimed at exploring the characteristics of CVI in a large sample of children affected by CP according to three different age groups (infants 6 months–2 years; pre-school age 3–5 years; school age > 6 years). Our hypothesis is that the clinical spectrum of CVI may vary according to age; particularly the older children may show a milder visual impairment characterized by a lower number of visual signs compared to the younger ones, due to visual system maturation and adaptive neuroplasticity that has implications in the organization of motor, somatosensory and visual functions (Fiori et al., 2019).

We found signs of CVI in a high percentage of children (180 out 193, 93%); this data confirms our previous study on children affected by CP (Fazzi et al., 2012) in which, for example, a reduced visual acuity was detected in 87% of the subjects and altered saccadic movements in 89%. We suggest that the higher percentage detected in the present study (compared to the one reported in literature, which ranges from 16 to 70%) is related to the evaluation method that involves a multidisciplinary team (comprising a child neuropsychiatrist, ophthalmologist, orthoptist, and child therapist specializing in visual function and neurological development). Furthermore, the video-recordings of the assessment allow the professionals to observe and judge the child performance at a later time (especially the qualitative functions as fixation, smooth pursuit and saccadic movements).

This study shows that refractive errors, especially astigmatism associated with hypermetropia or less frequently with myopia, are extremely common in children affected by CP. Our data were in line with literature (Jacobson and Dutton, 2000; Kozeis et al., 2007, 2015; Marasini et al., 2011; Fazzi et al., 2012; Park et al., 2016). It has been hypothesized that the preterm birth and postnatal distress or diseases can interfere with the normal emmetropisation process (Hsieh et al., 2012), especially in children with brain lesions (Sobrado et al., 1999; Saunders et al., 2010). Moreover, we observed that the high frequency of refractive errors persisted among the three age-subgroups although hypermetropia tended to decrease in contrast to the progression of myopia. These findings seem to be similar to those observed in healthy subjects. During childhood a lower percentage of hypermetropia and a higher percentage of myopia is observed due to intrinsic and extrinsic factors such as the fast progression and axial length elongation of eye as well as environment, particularly extensive near work (schooling, study, reading) that have been known to cause abnormal eye growth (Kaiti et al., 2021). The high prevalence of refractive errors in CP highlights the importance of screening for these easily treatable disorders since the first years of life. Indeed, uncorrected refractive errors can limit activities of daily living, impair the development of cognitive functions (Aghaji et al., 2013) and may increase the risk of reading difficulties (Kozeis et al., 2015).

Fundus oculi abnormalities were detected in more than half of children in each subgroup and no significant differences among the three groups were found. These abnormalities consist of optic disc pallor, isolated optic disc cupping and optic nerve hypoplasia and may be related to axonal loss due to brain damage (retrograde transynaptic degeneration) as reported by Jacobson et al. (1997) and in previous studies by our group (Ruberto et al., 2006; Fazzi et al., 2012).

We observed strabismus in almost three-quarters of children in each subgroup. Strabismus is common in children affected by CP (Fazzi et al., 2012) due to defects of afferent pathways caused by axonal interruption in the optic radiation (Jacobson and Dutton, 2000), abnormality of vergence neurons (Mays et al., 1986) or of the pathways involved in eye movements (Tychsen and Lisberger, 1986), maldevelopment/dysfunction of visual cortex (Tychsen et al., 1996). Esotropia was the most common type of strabismus in group 1 and 2 while exotropia in group 3. There are contrasting data on which type of ocular misalignment (esotropia vs. exotropia) is the most common in CP children (Collins, 2014; Park et al., 2016; Jeon et al., 2019; Duke et al., 2020). Several factors may be related to the expression of esotropia or exotropia, as the type and the severity of CP (Collins, 2014; Jeon et al., 2019), brain injury (Brodsky, 2016) or ethnicity (Duke et al., 2020). We hypothesized that another factor may be age, since data on healthy individuals report that esotropia occurs more frequently in infants and children at preschool age (Matsuo and Matsuo, 2005; Khorrami-Nejad et al., 2018).

Nystagmus, mostly represented by continuous jerk nystagmus or transient, and extrinsic ocular motility disorders, mostly characterized by abduction deficit, was detected in about half of the sample and the presence seemed to be stable with age. Different prevalences of nystagmus have been reported in literature, ranging from 1 to 50% in children affected by CP (Marasini et al., 2011; Fazzi et al., 2012; Dufresne et al., 2014; Tinelli et al., 2020). We suspected that our data were in the higher range probably because we considered all types of nystagmus (continuous and transient forms). Our data agreed with literature for the prevalence of extrinsic ocular motility disorders (Fazzi et al., 2012).

The most frequently oculomotor function anomalies detected were discontinuous smooth pursuit and impaired saccadic movements (mainly dysmetric with increased latency). Literature reports that the prevalence rate of oculomotor disorders in CP varies from 22 to 85% (for smooth pursuit) and from 18 to 89% (for saccades) (Fazzi et al., 2012; Duke et al., 2020). The presence of altered smooth pursuit and saccadic movements prevents children affected by CP from acquiring environmental scanning strategies and exploiting their visual function (Salati et al., 2002) and it can contribute to learning difficulties such as impaired reading (Jacobson et al., 1996). Comparing the subgroups, we observed a progressive improvement of fixation (from subgroup 1 to subgroup 2 and 3), smooth pursuit (from subgroup 1 to subgroup 3 and from subgroup 2 to subgroup 3) and saccadic movements (from subgroup 2 to subgroup 3) according to age. It is widely known that oculomotor functions progressively develop with age in healthy subjects (Helo et al., 2014). If the ability to fixate a target is already acquired in the first months of life (Chandna, 1991), the accuracy of smooth pursuit improves (Ross et al., 1993) and saccadic movements stabilize (Fukushima et al., 2000; Luna and Sweeney, 2004; Irving et al., 2006; Alahyane et al., 2016) until adolescence (Aring et al., 2007; Luna et al., 2008). However, data on the maturation processes of oculomotor functions in subjects with brain lesions are poor, but they could occur even in the presence of cerebral nervous system damage. Ego et al. (2015) showed that children with CP aged from 5- to 16-year-old present an improvement of oculomotor functions with age comparable to that of healthy subjects. A possible explanation may be that the participation in daily life activities spontaneously ameliorate oculomotor functions during childhood. In the present study we revealed that fixation improved from the preschool age while smooth pursuit and saccades needed more time to ameliorate; in fact, we noted a significant improvement during school age. This could be related to the fact that the ability to fixate is necessary to realize smooth pursuit or saccades, in fact eye movements depend not only on target-related signals from the peripheral visual field but also on the ability to fixate a target at the fovea (Krauzlis et al., 2017). Moreover, fixation is controlled by neuronal mechanisms that include many of the same brain regions involved in the generation of voluntary eye movements (superior colliculi and medio-posterior cerebellum). Early and accurate evaluation of oculomotor functions is therefore an important tool used to monitor the outcomes of dedicated training programs aimed at improving fixation time, pursuit movements and saccadic efficiency.

Our results also confirm the high frequency of basic visual function disorders in children affected by CP (Fazzi et al., 2012; Tinelli et al., 2020). Reduced visual acuity was detected in three quarters of the sample, limitation in visual field in almost half and altered contrast sensitivity in one third. Comparing the three subgroups, we found a progressive improvement of visual acuity and of contrast sensitivity (both from subgroup 1 to subgroup 2 and from subgroup 1 to subgroup 3) and of visual field (from subgroup 1 to subgroup 3) according to age. Literature on physiological maturation occurring in healthy new-borns suggests that perceptual visual abilities improve with age, especially during the first years of life due to visual system maturation characterized by the development of foveal cones and refinement of retinal and cortical architecture and to environmental factors (Lewis and Maurer, 2005; Sgandurra et al., 2017; Fazzi et al., 2021). Although studies on maturation of perceptual functions in subjects with brain injury are limited, they document an improvement of these skills. In a recent study of our group (Fazzi et al., 2021) a better visual acuity and contrast sensitivity has been documented not only in infants who underwent an early visual treatment but also in the control group. We hypothesized that the developing brain would be able to “amplify” visual function through neuroplastic changes involving local and global functional connectivity networks by activating, modulating and strengthening residual visual signals (Sabel et al., 2018; Fazzi et al., 2021). As regard visual field, some authors have observed a recovery of visual field limitation in children with early brain lesions at school age, probably attributable to the maturation of the ability to shift attention rather than an enlargement of the visual field (Mercuri et al., 2003; Guzzetta et al., 2010). MCA analysis confirmed our results, underlying the differences in the expression of CVI spectrum according to age: the younger children presented a wider association of signs of visual function involvement, while the older ones had a milder CVI phenotype consisting of limited number of visual dysfunctions. Unfortunately, the cognitive visual profile could not be included in the MCA because assessed only in children over 6 years of age.

More than half of children assessed for cognitive visual functioning belonging to subgroup 3 presented signs of CVDs, with visual motor and visual perception skills often simultaneously impaired. Specifically, visual motor abilities (BC and VMI) and perceptual categorization (UP and UL) seemed to be the most affected. These difficulties seem to be related to a damage to the superior longitudinal fasciculus, connecting the occipital cortex with the parietal-frontal cortices, as documented in our previous work (Galli et al., 2018). There is no accepted prevalence of these disorders among children with CP, with the rate found to vary between 5 and 85% (Stiers et al., 2002; Fazzi et al., 2004; Atkinson and Braddick, 2007; Pagliano et al., 2007; Ortibus et al., 2009; Ego et al., 2015) depending on the assessment tools applied, the diagnostic criteria used, and the type of CP evaluated. A positive association was found between FIQ/PIQ and CVDs, a pattern well documented in the literature (Ito et al., 1997; Fedrizzi et al., 1996; Stiers et al., 1999, 2002). However, literature data report that CVDs seem to be independent from impairment in non-verbal intelligence, reflecting the coexistence of two separate deficits (the so called, “dual deficit hypothesis”) (Stiers et al., 1999). To confirm the data and/or verify alternative hypothesis, it would be useful to expand the case series and consider other possible influencing factors, as the characteristics of brain lesions (type, timing, site), the clinical pictures (such as bilateral or unilateral CP), and the neurovisual profiles. Cognitive visual function involvement can be detected at school age, even when visual functions, such as visual acuity, are normal or only mildly impaired. In children over 6 years of age, these difficulties may cause problems in academic skills as mathematics and reading (Ben Itzhak et al., 2020). In this regard, literature data report that visual motor difficulties could be related to calculation problem solving (Arp et al., 2006) and operations as borrowing or carrying (Venneri et al., 2003) and that both the visual motor and perceptual abilities were involved in different level of visual word processing (Rosazza et al., 2009; Woolnough et al., 2021). Therefore, clinicians need to be aware of this possibility in order to early recognize a CVD and suggest environmental adaptation mitigating their impact on academic skills.

The main strength of the present study is that we did not use questionnaires or registers to collect data, instead we evaluated the children with CP directly using a complete and detailed video-recorded visual function examination, based on a multidisciplinary approach that involves the participation of several health professionals (child neuropsychiatrist, ophthalmologist, orthoptist, psychologist and child therapists specializing in visual function and neurological development).

Regarding potential limitations associated with the study, we need to consider that it was not conducted using a longitudinal design but instead selecting each subject that was consecutively referred to our Center. Hence, the risk of selection bias has to be considered. For this reason, we are carrying out a longitudinal study on 50 children affected by CP belonging to the sample presented in this work: preliminary data seem to confirm our findings (oculomotor functions seem to improve with age in more than half of the sample while basic visual functions in about one third of cases). Moreover, the cross-sectional design without normal controls should also be mentioned as limitation. Maturation effects may partly be caused by group differences since they were not matched; impairment rates (especially for the oculomotor functions) should be judged carefully in the absence of normative comparison. As regards the cognitive visual evaluation, we would mention two limitation. First, we could not investigate the presence of a cognitive visual dysfunction in all the children belonging to subgroup 3 due to the nature of the tests used for the assessment; it is highly likely that the children excluded from the cognitive visual assessment will have had significant cognitive visual deficits (with or without deficits in primary visual functions). Second, we cannot analyze the effect of age on CVDs since we were able to conduct this assessment only in those children over 6 years of age; further studies on characteristic of CVDs from pre-school age to adolescence should be performed considering also a larger sample size. Due to limited number of children assessed for the cognitive visual performances, the interpretation of our results needs attention and we cannot generalize them to all the sample.

On the basis of these observations, we can conclude that younger children with CP showed more signs of CVI compared to the older ones. In this direction, we suggest an early neurovisual evaluation for all these measures because it allows to define the type of habilitative intervention, directing resources toward the weak functions that can still improve. Acting as early as possible is fundamental given that neuroplasticity is maximal within the first 2 years of age (Yin et al., 2019). In our recent work we found that, although the presence of a spontaneous recovery, an early intervention could amplify the visual functions and the developmental outcomes (Fazzi et al., 2021), especially if the exposure to the visual training happens within the first years of life (for details on visual training please see Fazzi et al. (2021)). Moreover, an early assessment may prevent the mild spectrum of CVI from being unrecognized until it impacts on child’s learning, mobility, development, independence and quality of life, especially at school age (Bauer and Merabet, 2019).

We hypothesized that the improvement of visual functions could be related to the physiological maturation of the visual system and mechanisms of neuroplasticity that have induced the re-organization of visual functions after the damage (Ego et al., 2015). However, in contrast to the case of ocular blindness, literature data on morphological, structural and functional connectivity changes in subjects with CVI have been scant because highly heterogeneity across individuals in terms of location, timing, extent and cause of damage (Bennett et al., 2020). There is the need for further functional neuroimaging studies to investigate neural correlates associate with CVI and to the potential neuroplastic compensatory processes (Bennett et al., 2020).

## Data Availability Statement

The original contributions presented in the study are included in the article/Supplementary Material, further inquiries can be directed to the corresponding author/s.

## Ethics Statement

The studies involving human participants were reviewed and approved by the Comitato Etico di Brescia, ASST Spedali Civili di Brescia, Italy. Written informed consent to participate in this study was provided by the participants’ legal guardian/next of kin.

## Author Contributions

JG, EL, and AM drafted the manuscript. AM, AR, AF, and SM collected the data. SC performed the statistical analysis. JG, EF, and FS designed the study. All authors contributed to the article, reviewed the manuscript, and approved the submitted version.

## Conflict of Interest

The authors declare that the research was conducted in the absence of any commercial or financial relationships that could be construed as a potential conflict of interest.

## Publisher’s Note

All claims expressed in this article are solely those of the authors and do not necessarily represent those of their affiliated organizations, or those of the publisher, the editors and the reviewers. Any product that may be evaluated in this article, or claim that may be made by its manufacturer, is not guaranteed or endorsed by the publisher.

## References

[B1] Aghaji A. E. Lawrence L. Ezegwui I. Onwasigwe E. Okoye O. Ebigbo P. (2013). Unmet visual needs of children with down syndrome in an African population: implications for visual and cognitive development. *Eur. J. Ophthalmol.* 23 394–398. 10.5301/ejo.5000222 23335310

[B2] Alahyane N. Lemoine-Lardennois C. Tailhefer C. Collins T. Fagard J. Doré-Mazars K. (2016). Development and learning of saccadic eye movements in 7- to 42-month-old children. *J. Vis.* 16:6. 10.1167/16.1.6 26762275

[B3] Aring E. Grönlund M. A. Hellström A. Ygge J. (2007). Visual fixation development in children. *Graefes Arch. Clin. Exp. Ophthalmol.* 245 1659–1665. 10.1007/s00417-007-0585-6 17453232

[B4] Arp S. Taranne P. Fagard J. (2006). Global perception of small numerosities (subitizing) in cerebral-palsied children. *J. Clin. Exp. Neuropsychol.* 28 405–419. 10.1080/13803390590935426 16618628

[B5] Atkinson J. Braddick O. (2007). Visual and visuocognitive development in children born very prematurely. *Prog. Brain Res.* 164 123–149. 10.1016/S0079-6123(07)64007-2 17920429

[B6] Baranello G. Signorini S. Tinelli F. Guzzetta A. Pagliano E. Rossi A. (2020). Visual function classification system for children with cerebral palsy: development and validation. *Dev. Med. Child. Neurol.* 62 104–110. 10.1111/dmcn.14270 31180136

[B7] Bauer C. M. Merabet L. B. (2019). Perspectives on cerebral visual impairment. *Semin. Pediatr. Neurol.* 31 1–2. 10.1016/j.spen.2019.05.001 31548018

[B8] Beery K. E. Buktenica N. A. (2000). *VMI Developmental Test Of Visual-Motor Integration*, ed. Preda C. (Firenze: Giunti Os). Edizione Italiana A Cura Di.

[B9] Ben Itzhak N. Vancleef K. Franki I. Laenen A. Wagemans J. Ortibus E. (2020). Visuoperceptual profiles of children using the flemish cerebral visual impairment questionnaire. *Dev. Med. Child. Neurol.* 62 969–976. 10.1111/dmcn.14448 31889310

[B10] Bennett C. R. Bauer C. M. Bailin E. S. Merabet L. B. (2020). Neuroplasticity in cerebral visual impairment (CVI): assessing functional vision and the neurophysiological correlates of dorsal stream dysfunction. *Neurosci. Biobehav. Rev.* 108 171–181. 10.1016/j.neubiorev.2019.10.011 31655075 PMC6949360

[B11] Bova S. M. Fazzi E. Giovenzana A. Montomoli C. Signorini S. G. Zoppello M. (2007). The development of visual object recognition in school-age children. *Dev. Neuropsychol.* 31 79–102. 10.1207/s15326942dn3101_5 17305439

[B12] Brodsky M. C. (2016). Motion responses in human strabismus: what optokinesis in the deviating eye is telling us. *Invest. Ophthalmol. Vis. Sci.* 57:2990. 10.1167/iovs.16-19569 27273716

[B13] Castelli E. Fazzi E. (2016). SIMFER-SINPIA intersociety commission. recommendations for the rehabilitation of children with cerebral palsy. *Eur. J. Phys. Rehabil. Med.* 52 691–703. 26629842

[B14] Chandna A. (1991). Natural history of the development of visual acuity in infants. *Eye* 5(Pt. 1), 20–26. 10.1038/eye.1991.4 2060665

[B15] Collins M. L. (2014). Strabismus in cerebral palsy: when and why to operate. *Am. Orthopt. J.* 64 17–20. 10.3368/aoj.64.1.17 25313106

[B16] Donahue S. P. Baker C. N. Committee on Practice and Ambulatory Medicine, American Academy of Pediatrics, Section on Ophthalmology, American Academy of Pediatrics (2016). Procedures for the evaluation of the visual system by pediatricians. *Pediatrics* 137:e20153597. 10.1542/peds.2015-3597 26644488

[B17] Dufresne D. Dagenais L. Shevell M. I. REPACQ Consortium (2014). Spectrum of visual disorders in a population-based cerebral palsy cohort. *Pediatr. Neurol.* 50 324–328. 10.1016/j.pediatrneurol.2013.11.022 24468636

[B18] Duke R. E. Nwachukuw J. Torty C. Okorie U. Kim M. J. Burton K. (2020). Visual impairment and perceptual visual disorders in children with cerebral palsy in Nigeria. *Br. J. Ophthalmol.* Online ahead of print, 10.1136/bjophthalmol-2020-317768 33268343

[B19] Dutton G. N. (2003). Cognitive vision, its disorders and differential diagnosis in adults and children: knowing where and what things are. *Eye* 17 289–304. 10.1038/sj.eye.6700344 12724689

[B20] Ego C. Orban de Xivry J. J. Nassogne M. C. Yüksel D. Lefèvre P. (2015). Spontaneous improvement in oculomotor function of children with cerebral palsy. *Res. Dev. Disabil.* 36C 630–644. 10.1016/j.ridd.2014.10.025 25462523

[B21] Eliasson A. C. Krumlinde-Sundholm L. Rösblad B. Beckung E. Arner M. Ohrvall A. M. (2006). The Manual Ability Classification System (MACS) for children with cerebral palsy: scale development and evidence of validity and reliability. *Dev. Med. Child. Neurol.* 48 549–554. 10.1017/S0012162206001162 16780622

[B22] Fazzi E. Bova S. M. Uggetti C. Signorini S. G. Bianchi P. E. Maraucci I. (2004). Visual-perceptual impairment in children with periventricular leukomalacia. *Brain Dev.* 26 506–512. 10.1016/j.braindev.2004.02.002 15533651

[B23] Fazzi E. Micheletti S. Calza S. Merabet L. Rossi A. Galli J. (2021). Early visual training and environmental adaptation for infants with visual impairment. *Dev. Med. Child Neurol.* 63 1180–1193. 10.1111/dmcn.14865 34813110 PMC8518055

[B24] Fazzi E. Signorini S. G. Bova S. M. La Piana R. Ondei P. Bertone C. (2007). Spectrum of visual disorders in children with cerebral visual impairment. *J. Child. Neurol.* 22 294–301. 10.1177/08830738070220030801 17621499

[B25] Fazzi E. Signorini S. G. La Piana R. Bertone C. Misefari W. Galli J. (2012). Neuro-ophthalmological disorders in cerebral palsy: ophthalmological, oculomotor, and visual aspects. *Dev. Med. Child. Neurol.* 54 730–736. 10.1111/j.1469-8749.2012.04324.x 22712803

[B26] Fedrizzi E. Inverno M. Bruzzone M. G. Botteon G. Saletti V. Farinotti M. (1996). MRI features of cerebral lesions and cognitive functions in preterm spastic diplegic children. *Pediatr. Neurol.* 15 207–212. 10.1016/s0887-8994(96)00174-9 8916157

[B27] Fiori S. Staudt M. Boyd R. N. Guzzetta A. (2019). Neural plasticity after congenital brain lesions. *Neural Plast.* 2019:9154282. 10.1155/2019/9154282 31191640 PMC6525879

[B28] Fukushima J. Hatta T. Fukushima K. (2000). Development of voluntary control of saccadic eye movements. I. age-related changes in normal children. *Brain Dev.* 22 173–180. 10.1016/s0387-7604(00)00101-7 10814900

[B29] Galli J. Ambrosi C. Micheletti S. Merabet L. B. Pinardi C. Gasparotti R. (2018). White matter changes associated with cognitive visual dysfunctions in children with cerebral palsy: a diffusion tensor imaging study. *J. Neurosci. Res.* 96 1766–1774. 10.1002/jnr.24307 30027677

[B30] Green E. Stroud L. Bloomfield S. Cronje J. Foxcroft C. Hurter K. (2017). *Griffiths III. Griffiths Scales of Child Development*, Third Edn, eds Lanfranchi S. Rea M. Vianello R. Ferri R. (Firenze: Hogrefe). Edizione Italiana a cura.

[B31] Guzzetta A. D’acunto G. Rose S. Tinelli F. Boyd R. Cioni G. (2010). Plasticity of the visual system after early brain damage. *Dev. Med. Child Neurol.* 52 891–900. 10.1111/j.1469-8749.2010.03710.x 20561008

[B32] Guzzetta A. Fazzi B. Mercuri E. Bertuccelli B. Canapicchi R. van Hof-van Duin J. (2001). Visual function in children with hemiplegia in the first years of life. *Dev. Med. Child. Neurol.* 43 321–329. 10.1017/s0012162201000603 11368485

[B33] Handa S. Saffari S. E. Borchert M. (2018). Factors associated with lack of vision improvement in children with cortical visual impairment. *J. Neuroophthalmol.* 38 429–433. 10.1097/WNO.0000000000000610 29232345

[B34] Heersma D. J. van-Hof-Van Duin J. Hop W. C. J. (1989). Age norms for visual field development in children aged 0 to 4 years using arc perimetry. *Invest. Ophthalmol. Vis. Sci.* 30(Suppl.):242.

[B35] Helo A. Pannasch S. Sirri L. Rämä P. (2014). The maturation of eye movement behavior: scene viewing characteristics in children and adults. *Vision Res.* 103 83–91. 10.1016/j.visres.2014.08.006 25152319

[B36] Himmelmann K. Horber V. De La Cruz J. Horridge K. Mejaski-Bosnjak V. Hollody K. (2017). MRI classification system (MRICS) for children with cerebral palsy: development, reliability, and recommendations. *Dev. Med. Child Neurol.* 59 57–64. 10.1111/dmcn.1316 27325153

[B37] Hsieh C. J. Liu J. W. Huang J. S. Lin K. C. (2012). Refractive outcome of premature infants with or without retinopathy of prematurity at 2 years of age: a prospective controlled cohort study. *Kaohsiung J. Med. Sci.* 28 204–211. 10.1016/j.kjms.2011.10.010 22453068 PMC11916860

[B39] Hyvärinen L. Näsänen R. Laurinen P. (1980). New visual acuity test for pre-school children. *Acta Ophthalmol.* 58 507–511. 10.1111/j.1755-3768.1980.tb08291.x 7211248

[B40] Iodice A. Galli J. Molinaro A. Franzoni A. Micheli R. Pinelli L. (2018). Neurovisual assessment in children with ataxia telangiectasia. *Neuropediatrics* 49 26–34. 10.1055/s-0037-1607216 28992644

[B41] Irving E. L. Steinbach M. J. Lillakas L. Babu R. J. Hutchings N. (2006). Horizontal saccade dynamics across the human life span. *Invest. Ophthalmol. Vis. Sci.* 47 2478–2484. 10.1167/iovs.05-1311 16723459

[B42] Ismail F. Y. Fatemi A. Johnston M. V. (2017). Cerebral plasticity: windows of opportunity in the developing brain. *Eur. J. Paediatr. Neurol.* 21 23–48. 10.1016/j.ejpn.2016.07.007 27567276

[B43] Ito J. Araki A. Tanaka H. Tasaki T. Cho K. (1997). Intellectual status of children with cerebral palsy after elementary education. *Pediatr. Rehabil.* 1 199–206. 10.3109/17518429709167360 9689256

[B44] Jacobson L. K. Dutton G. N. (2000). Periventricular leukomalacia: an important cause of visual and ocular motility dysfunction in children. *Surv. Ophthalmol.* 45 1–13. 10.1016/s0039-6257(00)00134-x 10946078

[B45] Jacobson L. Hellström A. Flodmark O. (1997). Large cups in normal-sized optic discs: a variant of optic nerve hypoplasia in children with periventricular leukomalacia. *Arch. Ophthalmol.* 115 1263–1269. 10.1001/archopht.1997.01100160433007 9338671

[B46] Jacobson L. Ygge J. Flodmark O. (1996). Oculomotor findings in preterm children with periventricular leukomalacia. A connection between lesions in the periventricular area and eye motility disorders? *Acta Ophthalmol. Scand.* 74:645. 10.1111/j.1600-0420.1996.tb00755.x 9017062

[B47] Jeon H. Jung J. H. Yoon J. A. Choi H. (2019). Strabismus is correlated with gross motor function in children with spastic cerebral palsy. *Curr. Eye Res.* 44 1258–1263. 10.1080/02713683.2019.1631851 31189336

[B48] Kaiti R. Shyangbo R. Sharma I. P. Dahal M. (2021). Review on current concepts of myopia and its control strategies. *Int. J. Ophthalmol.* 14 606–615. 10.18240/ijo.2021.04.19 33875955 PMC8025164

[B49] Khorrami-Nejad M. Akbari M. R. Khosravi B. (2018). The prevalence of strabismus types in strabismic Iranian patients. *Clin. Optom.* 10 19–24. 10.2147/OPTO.S147642 30214338 PMC6095557

[B50] Korkman M. Kirk U. Kemp S. (2011). *NEPSY*, 2nd Edn. Firenze: Giunti OS.

[B51] Kovács I. Kozma P. Fehér A. Benedek G. (1999). Late maturation of visual spatial integration in humans. *Proc. Natl. Acad. Sci. U.S.A.* 96 12204–12209. 10.1073/pnas.96.21.12204 10518600 PMC18436

[B52] Kozeis N. Anogeianaki A. Mitova D. T. Anogianakis G. Mitov T. Klisarova A. (2007). Visual function and visual perception in cerebral palsied children. *Ophthalmic Physiol. Opt.* 27 44–53. 10.1111/j.1475-1313.2006.00413.x 17239189

[B53] Kozeis N. Panos G. D. Zafeiriou D. I. de Gottrau P. Gatzioufas Z. (2015). Comparative study of refractive errors, strabismus, microsaccades, and visual perception between preterm and full-term children with infantile cerebral palsy. *J. Child Neurol.* 30 972–975. 10.1177/0883073814549248 25296927

[B54] Kran B. S. Lawrence L. Mayer D. L. Heidary G. (2019). Cerebral/cortical visual impairment: a need to reassess current definitions of visual impairment and blindness. *Semin. Pediatr. Neurol.* 31 25–29. 10.1016/j.spen.2019.05.005 31548020

[B55] Krauzlis R. J. Goffart L. Hafed Z. M. (2017). Neuronal control of fixation and fixational eye movements. *Philos. Trans. R. Soc. Lond. B Biol. Sci.* 372:20160205. 10.1098/rstb.2016.0205 28242738 PMC5332863

[B56] Leat S. J. Wegmann D. (2004). Clinical testing of contrast sensitivity in children: age-related norms and validity. *Optom. Vis. Sci.* 81 245–254. 10.1097/00006324-200404000-00010 15097766

[B57] Lewis T. L. Maurer D. (2005). Multiple sensitive periods in human visual development: evidence from visually deprived children. *Dev. Psychobiol.* 46 163–183. 10.1002/dev.20055 15772974

[B58] Lewis T. L. Maurer D. (2009). Effects of early pattern deprivation on visual development. *Optom. Vis. Sci.* 86 640–646. 10.1097/OPX.0b013e3181a7296b 19417706

[B59] Luna B. Sweeney J. A. (2004). The emergence of collaborative brain function: FMRI studies of the development of response inhibition. *Ann. N. Y. Acad. Sci.* 1021 296–309. 10.1196/annals.1308.035 15251900

[B60] Luna B. Velanova K. Geier C. F. (2008). Development of eye-movement control. *Brain Cogn.* 68 293–308. 10.1016/j.bandc.2008.08.019 18938009 PMC2731686

[B61] Maioli C. Falciati L. Galli J. Micheletti S. Turetti L. Balconi M. (2019). Visuospatial attention and saccadic inhibitory control in children with cerebral palsy. *Front. Hum. Neurosci.* 13:392. 10.3389/fnhum.2019.00392 31780913 PMC6856641

[B62] Marasini S. Paudel N. Adhikari P. Shrestha J. B. Bowan M. (2011). Ocular manifestations in children with cerebral palsy. *Optom. Vis. Dev.* 42 178–182. 22187765

[B63] Matsuba C. A. Jan J. E. (2006). Long-term outcome of children with cortical visual impairment. *Dev. Med. Child Neurol.* 48 508–512. 10.1017/S0012162206001071 16700945

[B64] Matsuo T. Matsuo C. (2005). The prevalence of strabismus and amblyopia in Japanese elementary school children. *Ophthalmic Epidemiol.* 12 31–36. 10.1080/09286580490907805 15848918

[B65] Mays L. E. Porter J. D. Gamlin P. D. Tello C. A. (1986). Neural control of vergence eye movements: neurons encoding vergence velocity. *J. Neurophysiol.* 56 1007–1021. 10.1152/jn.1986.56.4.1007 3783225

[B66] Merabet L. B. Devaney K. J. Bauer C. M. Panja A. Heidary G. Somers D. C. (2016). Characterizing visual field deficits in cerebral/Cortical Visual Impairment (CVI) using combined diffusion based imaging and functional retinotopic mapping: a case study. *Front. Syst. Neurosci.* 10:13. 10.3389/fnsys.2016.00013 26941619 PMC4766290

[B67] Mercuri E. Anker S. Guzzetta A. Barnett A. Haataja L. Rutherford M. (2003). Neonatal cerebral infarction and visual function at school age. *Arch. Dis. Child. Fetal Neonatal Ed.* 88 F487–F491. 10.1136/fn.88.6.f487 14602696 PMC1763223

[B68] Ortibus E. Fazzi E. Dale N. (2019). Cerebral visual impairment and clinical assessment: the European perspective. *Semin. Pediatr. Neurol.* 31 15–24. 10.1016/j.spen.2019.05.004 31548019

[B69] Ortibus E. Lagae L. Casteels I. Demaerel P. Stiers P. (2009). Assessment of cerebral visual impairment with the L94 visual perceptual battery: clinical value and correlation with MRI findings. *Dev. Med. Child Neurol.* 51 209–217. 10.1111/j.1469-8749.2008.03175.x 19260932

[B70] Pagliano E. Fedrizzi E. Erbetta A. Bulgheroni S. Solari A. Bono R. (2007). Cognitive profiles and visuoperceptual abilities in preterm and term spastic diplegic children with periventricular leukomalacia. *J. Child Neurol.* 22 282–288. 10.1177/0883073807300529 17621497

[B71] Palisano R. Rosenbaum P. Walter S. Russell D. Wood E. Galuppi B. (1997). Development and reliability of a system to classify gross motor function in children with cerebral palsy. *Dev. Med. Child Neurol.* 39 214–223. 10.1111/j.1469-8749.1997.tb07414.x 9183258

[B72] Park M. J. Yoo Y. J. Chung C. Y. Hwang J. M. (2016). Ocular findings in patients with spastic type cerebral palsy. *BMC Ophthalmol.* 16:195. 10.1186/s12886-016-0367-1 27821110 PMC5100247

[B73] Pavlova M. A. Krägeloh-Mann I. (2013). Limitations on the developing preterm brain: impact of periventricular white matter lesions on brain connectivity and cognition. *Brain* 136(Pt. 4), 998–1011. 10.1093/brain/aws334 23550112

[B74] Philip S. S. Dutton G. N. (2014). Identifying and characterising cerebral visual impairment in children: a review. *Clin. Exp. Optom.* 97 196–208. 10.1111/cxo.12155 24766507

[B75] Philip S. S. Guzzetta A. Chorna O. Gole G. Boyd R. N. (2020). Relationship between brain structure and cerebral visual impairment in children with cerebral palsy: a systematic review. *Res. Dev. Disabil.* 99:103580. 10.1016/j.ridd.2020.103580 32004872

[B76] Rosazza C. Cai Q. Minati L. Paulignan Y. Nazir T. A. (2009). Early involvement of dorsal and ventral pathways in visual word recognition: an ERP study. *Brain Res.* 1272 32–44. 10.1016/j.brainres.2009.03.033 19332032

[B77] Ross R. G. Radant A. D. Hommer D. W. (1993). A developmental study of smooth pursuit eye movements in normal children from 7 To 15 years of age. *J. Am. Acad. Child Adolesc. Psychiatry* 32 783–791. 10.1097/00004583-199307000-00012 8340299

[B78] Ruberto G. Salati R. Milano G. Bertone C. Tinelli C. Fazzi E. (2006). Changes in the optic disc excavation of children affected by cerebral visual impairment: a tomographic analysis. *Invest Ophthalmol Vis Sci.* 47 484–488. 10.1167/iovs.05-0529 16431940

[B79] Sabel B. A. Flammer J. Merabet L. B. (2018). Residual vision activation and the brain-eye-vascular triad: dysregulation, plasticity and restoration in low vision and blindness - a review. *Restor. Neurol. Neurosci.* 36 767–791. 10.3233/RNN-180880 30412515 PMC6294586

[B80] Sakki H. E. Dale N. J. Sargent J. Perez-Roche T. Bowman R. (2018). Is there consensus in defining childhood cerebral visual impairment? A systematic review of terminology and definitions. *Br. J. Ophthalmol.* 102 424–432. 10.1136/bjophthalmol-2017-310694 29146757

[B81] Sakki H. Bowman R. Sargent J. Kukadia R. Dale N. (2021). Visual function subtyping in children with early-onset cerebral visual impairment. *Dev. Med. Child Neurol.* 63 303–312. 10.1111/dmcn.14710 33111315

[B82] Salati R. Borgatti R. Giammari G. Jacobson L. (2002). Oculomotor dysfunction in cerebral visual impairment following perinatal hypoxia. *Dev. Med. Child Neurol.* 44 542–550. 10.1017/s0012162201002535 12206621

[B83] Saunders K. J. Little J. A. McClelland J. F. Jackson A. J. (2010). Profile of refractive errors in cerebral palsy: impact of severity of motor impairment (GMFCS) and CP subtype on refractive outcome. *Invest. Ophthalmol. Vis. Sci.* 51 2885–2890. 10.1167/iovs.09-4670 20107180

[B84] Sgandurra G. Lorentzen J. Inguaggiato E. Bartalena L. Beani E. Cecchi F. (2017). A randomized clinical trial in preterm infants on the effects of a home-based early intervention with the ‘CareToy System’. *PLoS One* 12:e0173521. 10.1371/journal.pone.0173521 28328946 PMC5362053

[B85] Siu C. R. Murphy K. M. (2018). The development of human visual cortex and clinical implications. *Eye Brain* 10 25–36. 10.2147/EB.S130893 29760575 PMC5937627

[B86] Sobrado P. Suárez J. García-Sánchez F. A. Usón E. (1999). Refractive errors in children with cerebral palsy, psychomotor retardation, and other non-cerebral palsy neuromotor disabilities. *Dev. Med. Child Neurol.* 41 396–403. 10.1017/s0012162299000869 10400174

[B87] Stiers P. De Cock P. Vandenbussche E. (1999). Separating visual perception and non-verbal intelligence in children with early brain injury. *Brain Dev.* 21 397–406. 10.1016/s0387-7604(99)00050-9 10487474

[B88] Stiers P. Vanderkelen R. Vanneste G. Coene S. De Rammelaere M. Vandenbussche E. (2002). Visual-perceptual impairment in a random sample of children with cerebral palsy. *Dev. Med. Child Neurol.* 44 370–382. 10.1017/s0012162201002249 12088305

[B89] Street R. F. (1931). *A Gestalt Completion Contribution to Education.* New York, NY: Columbia University, Teachers College, Bureau of Publication.

[B90] Surveillance of Cerebral Palsy in Europe (2000). Surveillance of cerebral palsy in Europe: a collaboration of cerebral palsy surveys and registers. Surveillance of Cerebral Palsy in Europe (SCPE). *Dev. Med. Child Neurol.* 42 816–824. 10.1017/s0012162200001511 11132255

[B91] Teller D. Y. McDonald M. A. Preston K. Sebris S. L. Dobson V. (1986). Assessment of visual acuity in infants and children: the acuity card procedure. *Dev. Med. Child Neurol.* 28 779–789. 10.1111/j.1469-8749.1986.tb03932.x 3817317

[B92] Tinelli F. Guzzetta A. Purpura G. Pasquariello R. Cioni G. Fiori S. (2020). Structural brain damage and visual disorders in children with cerebral palsy due to periventricular leukomalacia. *Neuroimage Clin.* 28:102430. 10.1016/j.nicl.2020.102430 32980597 PMC7519396

[B93] Tychsen L. Lisberger S. G. (1986). Maldevelopment of visual motion processing in humans who had strabismus with onset in infancy. *J. Neurosci.* 6 2495–2508. 10.1523/JNEUROSCI.06-09-02495.1986 3746419 PMC6568682

[B94] Tychsen L. Burkhalter A. Boothe R. G. (1996) Funktionelle und strukturelle Abnormitäten im visuellen Cortex bei frühkindlichem Strabismus. *Klin Monbl. Augenheilkd.* 208, 18–22. 10.1055/s-2008-1035162 8839340

[B95] van Hof-van Duin J. Heersema D. J. Groenendaal F. Baerts W. Fetter W. P. (1992). Visual field and grating acuity development in low-risk preterm infants during the first 2 1/2 years after term. *Behav. Brain Res.* 49 115–122. 10.1016/s0166-4328(05)80201-3 1388794

[B96] Venneri A. Cornoldi C. Garuti M. (2003). Arithmetic difficulties in children with visuospatial learning disability (VLD). *Child Neuropsychol.* 9 175–183. 10.1076/chin.9.3.175.16454 13680407

[B97] Watson T. Orel-Bixler D. Haegerstrom-Portnoy G. (2007). Longitudinal quantitative assessment of vision function in children with cortical visual impairment. *Optom. Vis. Sci.* 84 471–480. 10.1097/OPX.0b013e31806dba5f 17568316

[B98] Wechsler D. (2002). *WPPSI: Technical and Interpretative Manual.* San Antonio, TX: The Psychological Corporation.

[B99] Wechsler D. (2003). *Wechsler Intelligence Scale for Children*, 4th Edn. San Antonio, TX: Harcourt Assessment, 10.1037/t15174-000

[B100] Wilson M. Quinn G. Dobson V. Breton M. (1991). Normative values for visual fields in 4- to 12-year-old children using kinetic perimetry. *J. Pediatr. Ophthalmol. Strabismus* 28 151–154. 1890572 10.3928/0191-3913-19910501-08

[B101] Woolnough O. Donos C. Rollo P. S. Forseth K. J. Lakretz Y. Crone N. E. (2021). Spatiotemporal dynamics of orthographic and lexical processing in the ventral visual pathway. *Nat. Hum. Behav.* 5 389–398. 10.1038/s41562-020-00982-w 33257877 PMC10365894

[B102] World Health Organization [WHO] (2021). *ICD-10: International Statistical Classification of Diseases and Related Health Problems : Tenth Revision*, 2nd Edn. Geneva: World Health Organization.

[B103] Yin W. Chen M. H. Hung S. C. Baluyot K. R. Li T. Lin W. (2019). Brain functional development separates into three distinct time periods in the first two years of life. *Neuroimage* 189 715–726. 10.1016/j.neuroimage.2019.01.025 30641240

