# Supplementary material for: Age-Related Effects on the Spectrum of Cerebral Visual Impairment in Children With Cerebral Palsy
Source: Front Hum Neurosci. 2022 Mar 2;16:750464. doi: 10.3389/fnhum.2022.750464 (PMC8924515; doi:10.3389/fnhum.2022.750464)
Supplement: Supplementary file 1 [file Data_Sheet_1.docx]

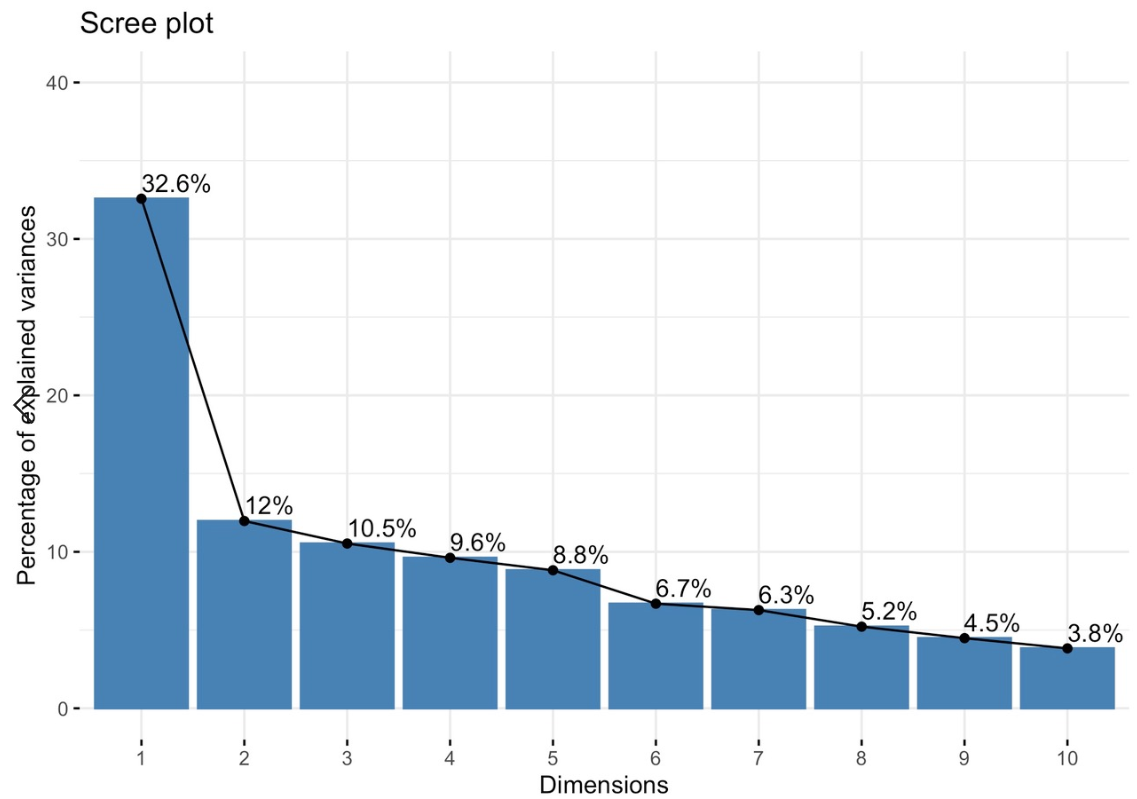


Figure S1: Scree plots of percentage of explained variances for dimensions established by the MCA with ten variables, identified from the neurovisual evaluation carried out in 180 children belonging to the three different age subgroups. Percentage of explained variances is a measure of the amount of variance accounted by every dimension produced by the MCA.
